# Identification of Potential Selective PAK4 Inhibitors Through Shape and Protein Conformation Ensemble Screening and Electrostatic-Surface-Matching Optimization

**DOI:** 10.3390/cimb47010029

**Published:** 2025-01-06

**Authors:** Xiaoxuan Zhang, Meile Zhang, Yihao Li, Ping Deng

**Affiliations:** 1 College of Pharmacy, Chongqing Medical University, Chongqing 400016, China; xiaoxuan_oxo@163.com (X.Z.); milezhang21@163.com (M.Z.); lee_xh500@hotmail.com (Y.L.); 2 Chongqing Research Center for Pharmaceutical Engineering, Chongqing 400016, China; 3 Chongqing Key Research Laboratory for Quality Evaluation and Safety Research of APIs, Chongqing 400016, China

**Keywords:** P21-activated kinases, selective inhibitors, molecular docking, molecular dynamics, MM/GBSA, electrostatic complementarity

## Abstract

P21-activated kinase 4 (PAK4) plays a crucial role in the proliferation and metastasis of various cancers. However, developing selective PAK4 inhibitors remains challenging due to the high homology within the PAK family. Therefore, developing highly selective PAK4 inhibitors is critical to overcoming the limitations of existing inhibitors. We analyzed the structural differences in the binding pockets of PAK1 and PAK4 by combining cross-docking and molecular dynamics simulations to identify key binding regions and unique structural features of PAK4. We then performed screening using shape and protein conformation ensembles, followed by a re-evaluation of the docking results with deep-learning-driven GNINA to identify the candidate molecule, STOCK7S-56165. Based on this, we applied a fragment-replacement strategy under electrostatic-surface-matching conditions to obtain Compd 26. This optimization significantly improved electrostatic interactions and reduced binding energy, highlighting its potential for selectivity. Our findings provide a novel approach for developing selective PAK4 inhibitors and lay the theoretical foundation for future anticancer drug design.

## 1. Introduction

P21-activated kinases (PAKs) are members of the serine/threonine protein kinase family and act as key downstream effectors of the small GTPases CDC42/RAC. They are involved in regulating cell growth and proliferation, and cytoskeletal remodeling [1,2]. The PAK family is divided into two groups: Group I (PAK1-3) and Group II (PAK4–6) [1]. PAK4 is highly associated with human cancers and promotes the proliferation and metastasis of various cancers, including ovarian [3], pancreatic [4,5,6], lung [7], and colorectal cancers [8,9], through its involvement in cytoskeletal remodeling and neurodevelopment [10]. These pathways are linked to cell growth and proliferation [11], making PAK4 a promising drug target. However, the highly homologous ATP-binding sites within the PAK family present a challenge in developing highly selective inhibitors targeting Group I or Group II PAKs [12]. Additionally, studies have shown that inhibiting Group I PAKs, particularly PAK1 and PAK2, increases cardiovascular toxicity [11]. Therefore, developing highly selective PAK4 inhibitors is crucial to mitigate the potential side effects associated with inhibiting Group I PAKs.

PAK4 inhibitors are broadly categorized into allosteric inhibitors and ATP-competitive inhibitors based on their mechanisms of action. Among them, KPT-9274 is the only allosteric inhibitor currently undergoing clinical trials; however, its specific inhibitory mechanism on PAK4 remains unclear. Additionally, KPT-9274 is a dual-target inhibitor acting on both PAK4 and NAMPT, which limits its selectivity towards PAK4 alone [13,14,15]. Consequently, developing ATP-competitive inhibitors with enhanced selectivity has become the primary focus. Examples include compounds such as PF-3758309 [16], KY-04031 [17,18], and LCH-7749944 [19] (Table S1). However, these inhibitors have encountered issues, including structural limitations, low kinase inhibitory activity, and poor subtype selectivity, ultimately leading to the termination of clinical trials. As a result, the pursuit of novel ATP-competitive PAK4 inhibitors with robust kinase inhibitory activity and high subtype selectivity remains of significant importance.

In this study, we aimed to evaluate the selectivity and binding affinity of candidate molecules by focusing on structural comparisons of PAK4 and binding pocket analysis, utilizing a combination of computational techniques such as virtual screening, molecular dynamics simulations, and re-scoring. Furthermore, we introduced a novel strategy for optimizing candidate molecules through fragment replacement combined with electrostatic surface matching. Additionally, methods such as independent gradient model based on Hirshfeld partition (IGMH) were used to more intuitively display our optimization results. This multidimensional approach provided a more reliable pathway for identifying potential highly selective and potent PAK4 inhibitors, addressing both efficacy and selectivity challenges.

## 2. Materials and Methods

**Figure 1:**
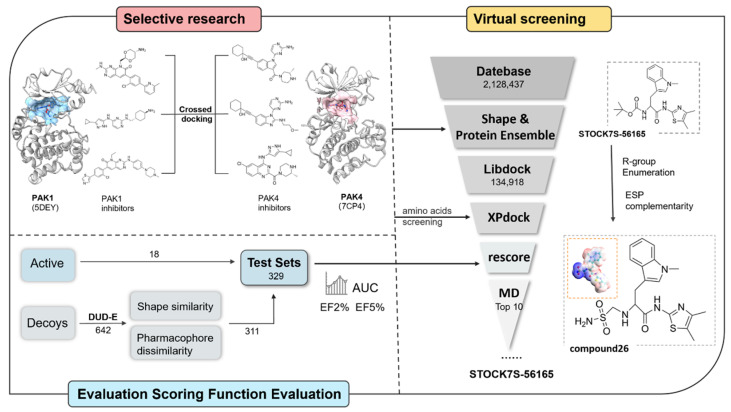
Overall workflow.

To better illustrate the complete process of the study, we have included a concise representation in Figure 1.

### 2.1. Protein and Ligand Preparation

The crystal structures of PAK1 (PDB ID: 5DEY) and PAK4 (PDB ID: 7CP4) were obtained from the RCSB PDB database (http://www.rcsb.org/ (accessed on 1 March 2023)). Using Schrödinger’s Protein Preparation Wizard [20], the protein structures were prepared for docking by removing water molecules, adding hydrogen atoms, and repairing the missing amino acid residues. The prepared protein structures then underwent energy minimization using the OPLS3 force field. Similarly, all small molecules were optimized and processed using Schrödinger’s LigPrep function under the OPLS3 force field to ensure they were ready for docking.

### 2.2. Cross-Docking

To further investigate the differences in selectivity between PAK1 and PAK4, we selected six representative selective inhibitors from the literature and the active compound database, which had complex crystal structures with PAK1 or PAK4 [21,22,23,24,25,26].

Cross-docking was used to identify differences between the active sites of PAK4 and PAK1. We extracted the six co-crystallized ligands from crystal complexes and cross-docked these inhibitors with the non-original subtype target using the XP protocol of Schrödinger’s Glide module. During the XP docking process, each small molecule generated up to 30 conformations, with the energy threshold set to 0.5 kcal/mol.

Based on the docking scores and binding modes, we selected the conformations of the PAK1 (PDBID: 5DEY) and PAK4 (PDBID: 7CP4) complexes with the highest docking scores as the reference systems for the next step of molecular dynamics (MD) simulations. The molecular dynamics analysis is shown in Figure S1.

### 2.3. Unbiased Molecular Dynamics Simulations

MD simulations and trajectory analysis were conducted using Gromacs 2021.6 [27]. Proteins were parameterized with the AMBER14ffSB force field [28], and simulations were performed in a rectangular solvent box (dimensions: 8.0 Å × 5.8 Å × 6.3 Å) filled with TIP3P water molecules [29]. The ligands were initially optimized using Gaussian09 [30] software at the B3LYP/def2tzvp level. Subsequent single-point energy calculations were then performed at the B3LYP-D3 (BJ)/ma-SVP level, and atomic RESP2 charges were calculated using Multiwfn 3.8 (dev) software [31]. The ligand force field parameters were derived from the general Amber force field (GAFF) [32], and the topology files for the ligand molecules were generated with the sobtop tool [33].

We added Cl^−^ to neutralize the charge of the protein–ligand complex system and the SHAKE [34] method was used to constrain bonds involving hydrogen atoms, with the simulation integration step set to 2 fs. Long-range electrostatic interactions were calculated using the PME [35,36] method, and periodic boundary conditions were employed to eliminate solvent box edge effects. The system energy was minimized to 100 kJ/mol without positional restraints. To maintain the temperature and pressure, velocity-rescale and Berendsen constant temperature and pressure simulations were conducted at 298.5 K and 1 bar, respectively, using the LINCS algorithm to fix covalent bonds involving hydrogen atoms. Each system then underwent an unconstrained MD simulation for 150 ns under constant temperature and pressure.

### 2.4. Binding Energy Calculation

Binding free energies and amino acid residue decomposition energies were calculated from the generated trajectories using the MM/GBSA method [37,38]. We extracted stable segments from 75 ns to 125 ns of the MD trajectory files for each system, with the exception of Compd 26, for which we selected the range from 100 ns to 250 ns. We sampled one frame every 50 ps for all systems, resulting in 1000 frames per system. For Compd 26, we adjusted the frame interval to 150 ps, resulting in 1000 frames for this compound as well, due to the extended time range. The print_res parameter was adjusted to calculate the energy of amino acids within 4 Å of the ligand, while other parameters were left at their default settings in the MMGBSA.py script. The formulas for calculating the binding free energy of the ligand with the protein receptor were as follows:


∆*G*_total_ = ∆*H* − T∆*S* = ∆*G*_GAS_ + ∆*G*_solv_ − T∆*S* (1)

∆*G*_GAS_ = ∆*G*_ele_ + ∆*G*_vdW_ (2)

∆*G*_solv_ = ∆*G*_GB_ + ∆*G*_surf_ (3)

In formula (1), the binding free energy (∆*G*_total_) is decomposed into gas-phase free energy (∆*G*_GAS_) and solvation free energy (∆*G*_solv_). The entropy effect (T∆*S*) is typically neglected because the conformational changes before and after binding are minimal when calculated by the MM/GBSA method, which offsets this contribution in the difference calculation. In formulas (2) and (3), Δ*G*_GAS_ is the sum of the electrostatic energy (Δ*G*_ele_) and the van der Waals energy (Δ*G*_vdw_). Δ*G*_solv_ represents the solvation free energy, which includes both polar solvation free energy (Δ*G*_GB_) and non-polar solvation free energy (Δ*G*_surf_), reflecting the solvation effects on ligand and receptor binding.

To further investigate the binding selectivity of inhibitors for PAK4 and PAK1, we calculated the free energy decomposition of residues within 4 Å of the ligand for both targets. By comparing the reference complex systems with the docked systems, we identified differences in key amino acid residues between the two subtypes that are critical for activity.

### 2.5. Shape Screening

A compound library containing 2,128,427 compounds was constructed by collecting and cleaning invalid structures from the Analyticon Discovery and Topscience databases. The Rdkit [39] package was used to perform initial optimization on all structures for shape-based screening.

Using Schrödinger’s shape-screening function, we set Compd 55 as the reference shape and conducted a shape alignment on 2,128,437 molecules from the Analyticon Discovery and Topscience databases. The screening threshold was set to 0.6, with a limit on the maximum number of retained hits to one-tenth of the total database size. Consequently, around 210,000 molecules with a similarity of above 60% were selected and imported into the Ligprep module to optimize structures using the same settings as previously employed.

### 2.6. Virtual Screening Based on Protein Conformational Ensembles

Based on the molecular dynamics trajectory of the PAK4 (PDB ID: 7CP4) complex structure, we performed conformational clustering on the trajectory using the gmx cluster module in GROMACS. The last 100 ns of the trajectory were used for clustering, with a cutoff set to 0.8. Three representative conformations were selected from the trajectory for virtual screening, with only the common screening results retained. For the candidates obtained from shape-based screening, we performed fast and high-precision screening using the LibDock module in Discovery Studio 2019 and the Glide_XP module in Schrödinger software, respectively.

### 2.7. Re-Scoring of Docking Conformation

To enhance the innovation and accuracy of virtual screening, we designed an evaluation framework to assess the performance of different scoring functions. First, we optimized the test set to ensure the reliability and accuracy of the study. All active compounds were carefully selected PAK4-selective inhibitors (see references and Table S1). Building on the previous shape-based screening, we improved the DUD-E model [40] (https://dude.docking.org/generate (accessed on 22 July 2023)) to eliminate false positives resulting from shape similarity. The process consisted of two steps: first, shape-based screening ensured that the decoys were structurally similar to the active compounds but lacked key functional features; second, ligand pharmacophore mapping was used to select 3 to 10 structurally similar but pharmacophore-differentiated decoys for each active compound. The final test set contained 329 molecules, including 18 active inhibitors and 311 decoys.

The test set molecules were docked to the PAK4 structure (PDB ID: 7CP4) using consistent docking parameters to ensure comparability. To evaluate the predictive power and accuracy of different scoring algorithms, we employed a diverse range of scoring functions, including machine-learning-based models such as RF_VS [41,42,43,44] and GNINA [45,46] (CNN_affinity, CNN_score, affinity, CNN_VS). Additionally, we incorporated traditional scoring methods from Discovery Studio, including PLP1, PLP2, LigScore1, LigScore2, Jain, PMF, and PMF04, as well as Schrödinger’s XP docking scoring functions (docking score, XP score, Glide score). Notably, the CNN_VS scoring function was derived as the product of CNN_affinity and CNN_score, providing a hybrid approach that integrated both machine learning and traditional scoring elements.

We assessed the performance of these scoring functions by calculating the receiver operating characteristic (ROC) curves and the area under the curve (AUC) for each function. Additionally, we calculated enrichment factors (*EF*s) for the top 2% and 5% of molecules in the test set to measure the scoring functions’ ability to enrich active compounds. Enrichment factor (*EF*) is defined by the following formula:

(4) $$EF=\frac{{hit}_{\left(sample\right)}}{N_{\left(sample\right)}}÷\frac{{hit}_{\left(all\right)}}{N_{\left(all\right)}}$$

where, *hit*_(sample)_/*N*_(sample)_ represents the proportion of active molecules in the top-scoring subset (2% or 5%) of the test set, while *hit*_(all)_/*N*_(all)_ represents the proportion of active molecules in the entire test set. This metric evaluates the degree to which scoring functions prioritize active molecules over decoys, providing a quantitative comparison of their effectiveness in virtual screening.

This multi-faceted evaluation not only showcases the strength of machine-learning-based scoring functions like CNN_VS but also emphasizes the novel approach of integrating pharmacophore mismatching decoys, enhancing the depth and rigor of virtual screening methodologies.

### 2.8. Molecular Optimization and Analysis

The RESP charges of the compounds were calculated at the B3LYP/6-311+G(d,p) theory level, and the electrostatic potential surface (ESP) was generated using Multiwfn. Fragment-replacement screening of the tert-butoxycarbonyl group in STOCK7S-56165 was performed using Schrödinger’s custom R-group counting function, resulting in the generation of 1985 new derivatives. IGMH [47] was conducted using Multiwfn, and the isosurfaces were visualized at a 0.005 contour level using VMD [48].

## 3. Results

### 3.1. Structural Comparison and Binding Site Analysis of PAK1 and PAK4

**Figure 2:**
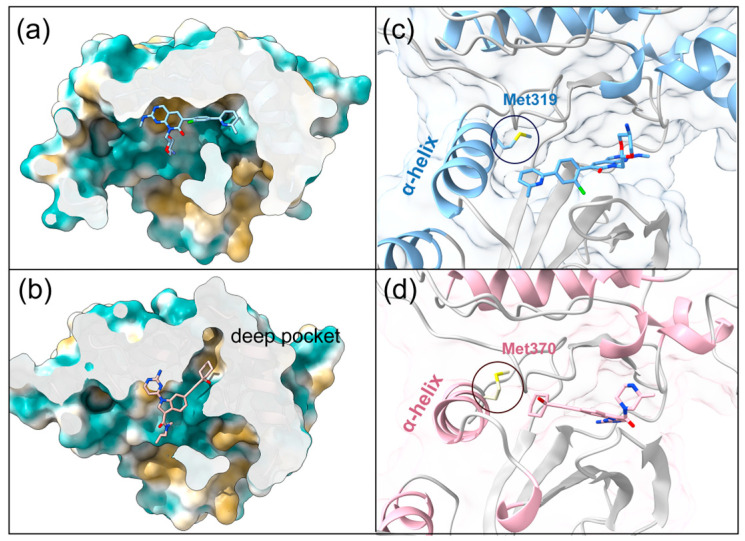
Structural comparison of the binding cavities in PAK1 and PAK4 with their ligands. (**a**) Crystal structure of PAK4 (PDB ID: 7CP4) and (**b**) crystal structure of PAK1 (PDB ID: 5DEY). The hydrophobic surfaces of the binding cavities are visualized using color shading. The secondary structures of the receptors, including the α-helices, are highlighted: PAK1 is shown in blue (**c**) and PAK4 is shown in pink (**d**).

Research has identified subtle differences in the shape of the active sites of PAK1 and PAK4, primarily due to changes in the orientation of the αC helix between the two proteins [21]. For example, in PAK1, the rotation of the αC helix positions the Met319 residue (equivalent to Met370 in PAK4) toward the pocket, occupying part of the space and narrowing one side of the hydrophobic pocket. In contrast, in PAK4, the Met370 residue points in the opposite direction, resulting in a deeper pocket with a slightly altered orientation (Figure 2). These differences in pocket shape provide potential opportunities to better accommodate and selectively target the PAK4 active site, thereby enhancing selectivity.

**Figure 3:**
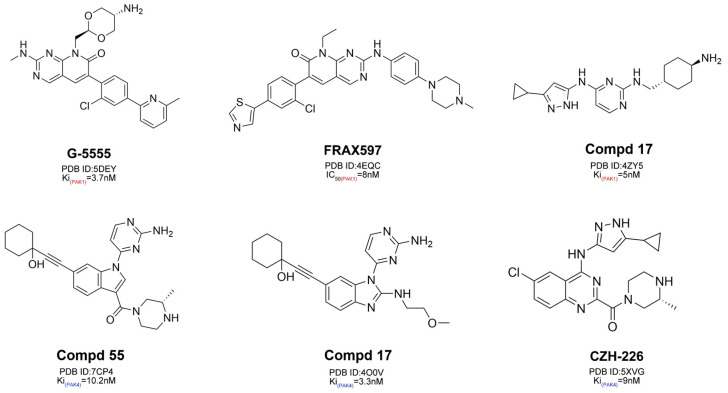
Chemical structures of representative inhibitors targeting PAK1 and PAK4.

**Figure 4:**
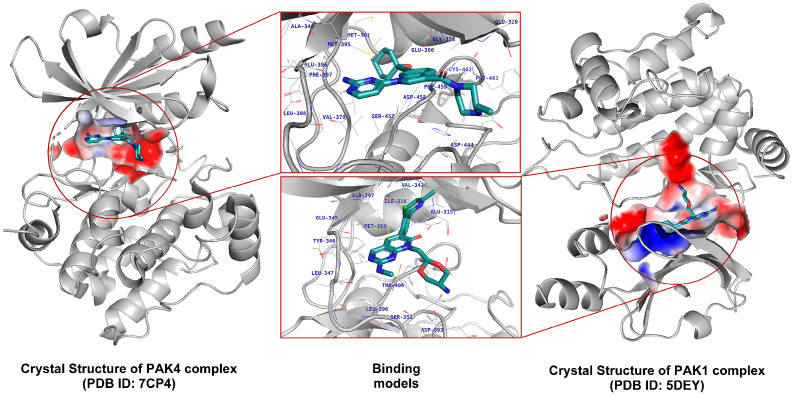
Binding cavities of PAK1 and PAK4. Left: Crystal structure of the PAK4 (PDB ID: 7CP4). Right: Crystal structure of the PAK1 (PDB ID: 5DEY). The binding cavities of both receptors are highlighted using electrostatic potential coloring, with key binding site residues labeled in blue.

**Figure 5:**
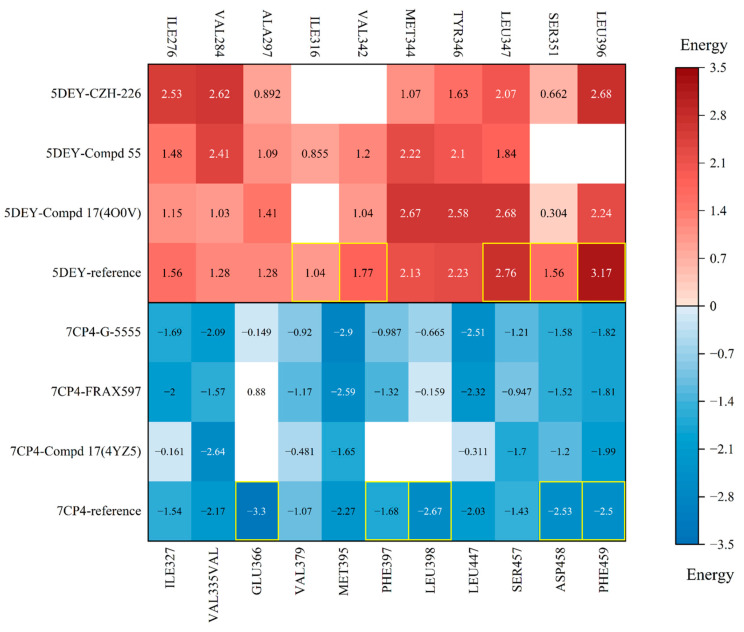
Heatmap of the free energy decomposition for PAK1 and PAK4 systems. Red indicates the interactions of inhibitors with key residues around the binding site in PAK1, while blue indicates the interactions of inhibitors with key residues around the binding site in PAK4. Key residues are highlighted in yellow boxes.

To further understand the differences in pocket characteristics, we first selected six representative inhibitor–complex crystal structures from the PDB database and identified the structural targets, PAK1 (PDB ID: 5DEY) and PAK4 (PDB ID: 7CP4), through cross-docking (Figure 3 and Figure 4). Subsequently, we identified the hotspot residues in the binding pockets by decomposing the binding free energies of each residue (Table S2) and visualized the results using a heatmap (Figure 5).

The heatmap shows the interaction strength with amino acid residues, where darker colors represent greater energy contributions. Residues more than 4 Å away from the ligand are displayed in white. The red group illustrates the energy decomposition of amino acid residues when PAK4 inhibitors bind to PAK1. In the same column, the reference row (PAK1 inhibitor in PAK1 docking system), the heatmap shows darker colors at residues LEU396, SER351, LEU347, VAL342, and ILE316, indicating that these residues play a more critical role in the binding of PAK1 with its inhibitors. Similarly, the blue group depicts the energy decomposition of amino acid residues when PAK1 inhibitors bind to PAK4. In the reference structure, it is clear that the residues GLU366, PHE397, LEU398, ASP458, and PHE459 in PAK4 play a crucial role in binding, particularly ASP458, which has been previously reported as a key residue influencing activity. All of these residues are located in the hydrophobic pockets on either side and within the ASP-PHE-GLY (DFG) motif.

### 3.2. Virtual Screening Based on Shape and Protein Conformational Ensembles

It has been demonstrated that shape-based screening methods can effectively enrich active molecules in virtual screening, particularly for large compound libraries containing millions of compounds [49]. Therefore, in this study, to improve screening efficiency, we first selected Compd 55 as the template for shape similarity screening (Figure 2), as its alkyl side chain effectively reaches the key hydrophobic pocket and forms stable interactions with critical residues such as GLU366, GLU396, and LEU398 in the hinge region and hydrophobic pocket [22]. Before formal docking, some unreasonable structures were preliminarily excluded, leaving 210,000 molecules for the next step of formal docking.

It is also worth noting that the intrinsic flexibility of proteins allows them to adopt multiple conformations under different physiological conditions, and this dynamic characteristic is often overlooked in traditional docking simulations, making it difficult to predict reliable binding modes of ligands [50,51,52]. This flexibility presents a major challenge for structure-based drug design, as proteins are not static structures, and the active sites in different conformations may accommodate different ligands. For example, when the active site expands, larger ligands are more likely to bind, while smaller ligands may fit better when the site contracts [52,53]. Crystal structures only reflect the protein state at a specific time point and are unable to fully capture its dynamic nature. Therefore, we obtained an ensemble of PAK4 protein structures through 150 ns molecular dynamics simulations and used clustering to identify representative conformations of the PAK4 protein for subsequent virtual screening.

Subsequently, we performed rapid docking screening of the molecules obtained in the previous step using the representative conformations of the PAK4 protein structure ensemble in Discovery Studio’s LibDock module. This helped further filter out unsuitable candidate molecules and focus on their interactions with the critical amino acid, ASP458 [16,21,22,54]. From these preliminary results, we retained compounds that exhibited at least one favorable interaction with ASP458, including hydrogen bonds and hydrophobic interactions, reducing the compound library to 134,918 molecules. We then performed high-precision docking of these selected compounds using the XP protocol of the Schrödinger Glide module, ultimately obtaining 26,423 candidate molecules for the next step of screening.

### 3.3. Re-Scoring to Improve Screening Performance

Re-scoring the docking poses of screened molecules can lead to better virtual screening performance compared with relying solely on the scoring functions used by traditional docking programs [55,56]. To improve the accuracy of virtual screening results, we evaluated a series of scoring functions using a well-established test set. The ROC curve is shown in Figure S2, and the AUC, EF2%, and EF5% metrics are presented in Table 1. According to the area under the ROC curve (AUC), the Lig2Score function (DS) exhibited the highest AUC, indicating its excellent ability to distinguish between active and inactive molecules. However, in actual virtual screening, only the top few molecules were retained from large databases. In this context, CNN_affinity and CNN_VS (GNINA) showed better performance. This suggests that data-driven deep-learning models can enhance the screening capability of scoring functions in virtual screening. Furthermore, when comparing the EF2% and EF5% results, we found that CNN_VS was more effective than CNN_affinity in enriching active molecules at the top of the list. Therefore, we selected CNN_VS as the final scoring function.

**Figure 6:**
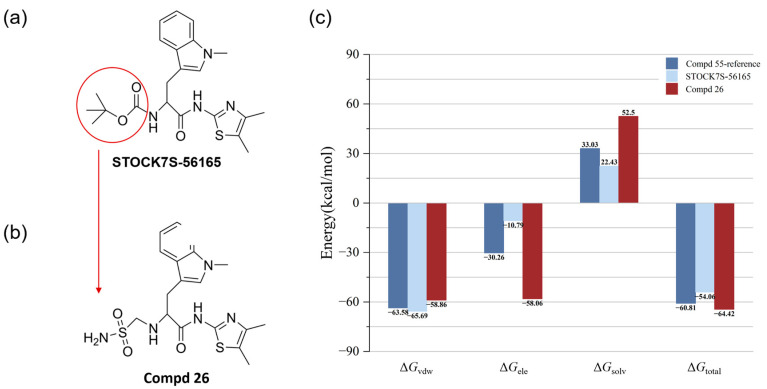
(**a**) The chemical structure of STOCK7S-56165, (**b**) the chemical structure of Compd 26, and (**c**) binding free energy contributions of Compd 55, STOCK7S-56165, and Compd 26 to PAK4 (energy unit: kcal/mol). The result for Compd 26 is the average value of stable 100-250 ns from three independent replicate simulations.

Next, we re-scored the protein–ligand complex structures of the 26,423 candidate molecules from the virtual screening using the CNN_VS function. We then selected the top 10 ligands with the highest scores from the MD simulations (Figure S3) and calculated their binding free energies (Table 2). The structures of the top-10-hit compounds with the highest scores from the MD simulations are shown in Figure S4, while Figure S5 illustrates their interactions with the PAK4 active site. The results showed that the compound STOCK7S-56165 (Figure 6a) exhibited a strong binding affinity for the PAK4 target, adopting a binding mode similar to that of Compd 55. However, compared with the original ligand, STOCK7S-56165 showed slightly enhanced van der Waals interactions, while its electrostatic interaction energy was significantly reduced. This reduction may have been a key factor affecting its overall binding energy.

### 3.4. Optimization of Candidate Molecules by Electrostatic Surface Matching Combined with Fragment Substitution

Research has shown that optimizing electrostatic surface matching is a key strategy in structure-based drug discovery, as it can enhance the affinity between molecules and specific protein targets [57]. Therefore, we calculated the electrostatic surface, which is closely related to electronic properties, and improved binding affinity and target selectivity by optimizing the electrostatic interactions between the ligand and the protein.

We compared the electrostatic surface potential (ESP) of STOCK7S-56165 with that of the 7CP4 active site and observed charge differences near the opening of the binding pocket, which may be one of the main reasons for the lower electrostatic energy. We then performed fragment-replacement screening using Schrödinger, resulting in 1985 new derivatives.

**Figure 7:**
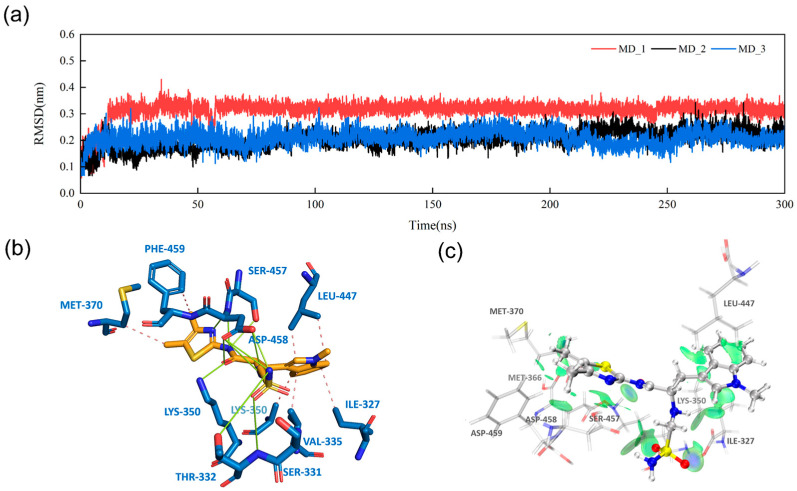
(**a**) RMSD analysis of Compd 26 during MD simulations from three independent replicate calculations, (**b**) 3D binding pose of Compd 26 with PAK4, where hydrogen bonds and hydrophobic interactions are represented by green and pink lines, respectively, and (**c**) IGMH analysis of the interaction between Compd 26 and PAK4; the green color block indicates that the main interaction is van der Waals interaction.

**Figure 8:**
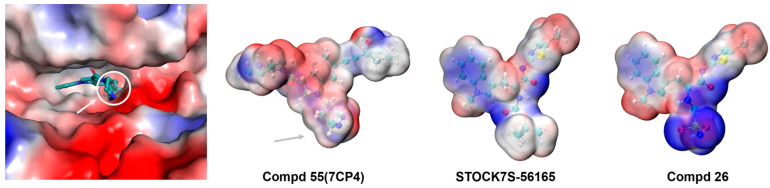
ESP surface of PAK4-binding site (PDB ID: 7CP4), Compd 55, STOCK7S-56165, and Compd 26.

Next, based on docking scores, MD simulations, and electrostatic-surface-matching calculations, we identified a new compound, Compd 26 (Figure 6b), from the top nine compounds (Figure S6). MD simulations of PAK4 with Compd 26 showed more stable behavior during the simulation compared with the other eight candidate compounds (Figure 7a and Table S3). Figure S7 illustrates the MD_3 trajectory, and similar trends were observed in the MD_1 and MD_2 simulations, which suggests that Compd 26 reached a stable conformation in PAK4 by 100 ns, with no significant changes in binding pose or interactions thereafter. When compared with Compd 55 and STOCK7S-56165 (Figure 6a), Compd 26 exhibited significant improvements in binding energy and electrostatic energy. ESP surface calculations revealed that replacing the group in the circular region with a sulfonamide group achieved optimal electrostatic surface matching at the opening of the PAK4 protein active site (Figure 8). Additionally, MM/GBSA results demonstrated significant improvements in both electrostatic energy and total binding energy (Figure 6c). These findings not only highlight the importance of electrostatic interactions but also provide new structural insights and opportunities for the discovery of PAK4 inhibitors.

### 3.5. Intermolecular Interactions and IGMH Analysis

To further understand the role of key amino acid residues in the interaction between Compd 26 and PAK4, we performed molecular dynamics simulations (Figure 7a) and PLIP [58] analysis to investigate the hydrogen bonds and hydrophobic interactions between Compd 26 and PAK4. The hydrogen bond occupancy clearly indicated that, compared with Compd 55, STOCK7S-56165 exhibited poorer hydrogen bond stability with key amino acids, which may be another reason for its lower binding energy (Table S4).

However, after replacing the group with a sulfonamide group, small changes in atom types and docking shape significantly increased the number of hydrogen bond donors and acceptors, leading to a substantial increase in the number of hydrogen bonds formed. Additionally, during the simulation, the 7CP4–compd26 complex formed strong hydrogen bonds with SER457, ASP458, THR332, LYS350, and SER331, with hydrogen bonds persisting for more than 20% of the simulation time. The hydrophobic regions containing these amino acids and the DFG motif are crucial for PAK4 activity (Table S4).

To further analyze the weak interactions between Compd 26 and PAK4, we conducted an IGMH analysis [47] to study the weak interactions between Compd 26 and STOCK7S-56165 at the PAK4 active site with key pocket residues (Figure 7b). Compared with STOCK7S-56165, Compd 26 showed weaker interactions at various contact points with the active site, achieving van der Waals energies similar to the template molecule, which was confirmed by MM/GBSA calculations.

The molecular dynamics results further supported these findings (Figure 6c). The calculated binding free energies (Δ*G*_total_) revealed that Compd 26 had a stronger overall binding affinity (−64.42 kcal/mol) compared with STOCK7S-56165 (−54.06 kcal/mol) and the template molecule (−60.81 kcal/mol). This was primarily driven by its significantly stronger electrostatic interaction energy (Δ*G*_ele_ = −58.06 kcal/mol), although it exhibited a higher solvation penalty (Δ*G*_solv_ = 52.5 kcal/mol) compared with the other compounds. These values for Compd 26 were derived from the average of three independent replicate simulations, as detailed in Table S5. These results underscore the potential of Compd 26 as a lead molecule, offering a balance between electrostatic and van der Waals interactions while maintaining favorable overall binding energy.

The protein–ligand complex binding mode (Figure 7b) and IGMH analysis results (Figure 7c) suggest that Compd 26 occupies and deeply extends into the PAK4 binding pocket. Docking results (Figure S8) confirmed that Compd 26 exhibits a binding mode similar to that of Compd 55 at the PAK4 active site, supporting its role as a PAK4 inhibitor. Furthermore, by replacing parts that conflict with the pocket surface charges, Compd 26 increased both electrostatic energy and the number of hydrogen bonds, leading to better matching with the PAK4 active site and enhanced binding affinity.

To investigate the selectivity of Compd 26 for PAK1 and PAK4, we used the XP protocol of Schrödinger’s Glide module to dock Compd 26 into both PAK1 and PAK4. The docking results were in line with our expectations. As shown in Figure S9 and the docking scores in Table S6, Compd 26 exhibited a significantly higher docking score with PAK4, and its dimethylthiazole fragment extended into the deep pocket of PAK4. This suggests that Compd 26 has stronger selectivity for binding to PAK4, indicating its potential as a selective PAK4 inhibitor.

## 4. Discussion

This study successfully screened and optimized potential high-selectivity inhibitors for PAK4 through structural analysis and computational screening. By comparing the structural features of the active sites of PAK1 and PAK4, we identified key differences in the binding pockets, providing an effective starting point for designing selective PAK4 inhibitors. Through virtual screening and subsequent re-scoring with deep-learning models, we identified candidate molecules that formed stable interactions with key PAK4 residues. Additionally, further optimization through fragment replacement and electrostatic surface matching significantly enhanced the electrostatic complementarity and binding affinity of the candidate molecules.

The final optimized compound, Compd 26, exhibited excellent theoretical selectivity and inhibitory activity for PAK4, laying a solid foundation for subsequent experimental validation and potential drug development. The methodological framework proposed in this study shows great promise in improving drug target selectivity and potency, providing an important reference for future anticancer drug design.

## Figures and Tables

**Table 1:** Performance metrics (AUC, EF2%, EF5%) for the evaluated scoring functions.

	EF_2%_	EF_5%_	AUC
**RF-VS**	10.45	8.00	0.86
**CNNaffinity**	18.28	7.80	0.83
**CNNscore**	15.67	9.14	0.71
**Affinity**	10.45	4.57	0.80
**CNN_VS**	18.28	8.00	0.72
**DockingScore**	15.67	8.00	0.82
**XPscore**	15.67	9.14	0.83
**GlideScore**	15.67	8.00	0.83
**Lig1**	7.83	4.57	0.80
**Lig2**	15.67	10.28	0.87
**PLP1**	15.67	7.80	0.86
**PLP2**	15.67	9.14	0.86
**Jain**	10.45	5.71	0.85
**PMF**	13.06	8.00	0.87
**PMF04**	0	1.14	0.72

**Table 2:** Binding free energy contributions of the top 10 to PAK4 (energy unit: kcal/mol).

Compounds	MM/GBSA				GNINA (CNN_VS)
	Δ*G*_vdw_	Δ*G*_ele_	Δ*G*_GB_	Δ*G*_total_	
**Compd 55**	−63.58	−30.26	39.99	−60.81	7.61
**HIT213882013**	−49.18	−19.88	29.30	−46.07	7.50
**STOCK1S-85434**	−66.13	−18.27	40.60	−51.98	7.63
**HIT104079502**	−51.46	−18.26	35.34	−40.69	7.60
**SN0341269**	−52.33	−17.66	42.92	−34.12	7.60
**STOCK7S-56165**	−65.69	−10.79	29.69	−54.06	7.58
**HIT212577525**	−53.27	−38.81	47.34	−51.10	7.47
**HIT105326727**	−51.16	−38.85	55.42	−41.80	7.46
**HIT105409527**	−45.53	−28.69	46.82	−33.59	7.41
**HIT213881679**	−58.67	−40.38	58.04	−47.43	7.36
**HIT104998753**	−52.48	−32.36	43.86	−47.41	7.35

## Data Availability

All data supporting the conclusions of this article are included in this article.

## Supplementary Materials

The following supporting information can be downloaded at: https://www.mdpi.com/article/10.3390/cimb47010029/s1.
