# Supplementary material for: Identification of Potential Selective PAK4 Inhibitors Through Shape and Protein Conformation Ensemble Screening and Electrostatic-Surface-Matching Optimization
Source: Curr Issues Mol Biol. 2025 Jan 6;47(1):29. doi: 10.3390/cimb47010029 (PMC11764389; doi:10.3390/cimb47010029)
Supplement: Supplementary file 1 [file cimb-47-00029-s001.zip › cimb-3361536-supplementary.pdf]

Supporting Information

Identification of Potential Selective PAK4 Inhibitors through Shape and Protein Conformation Ensemble Screening and Electrostatic Surface Matching Optimization

***Xiaoxuan Zhang*[1,2,†], *Meile Zhang*[1,2,†], *Yihao Li*[1,2], *Ping Deng*[1,2,3,*]**

[1] *College of Pharmacy, Chongqing Medical University, Chongqing, 400016, China.*

[2] *Chongqing Research Center for Pharmaceutical Engineering, Chongqing, 400016, China.*

[3] *Chongqing Key Research Laboratory for Quality Evaluation and Safety Research of APIs, Chongqing, 400016, China.*

† These authors contributed equally to this work.

*Correspondence and requests for materials should be addressed to Ping Deng(email: 100865@cqmu.edu.cn)

Table of contents

Supplementary Figures

Figure S1 RMSD analysis of co-crystal inhibitors during MD simulations after cross-docking. 3

Figure S2 ROC curves for the scoring functions

Figure S3 RMSD analysis of the top 10 hit compounds during MD simulations, excluding STOCK7S-56165. 3

Figure S4 Structures of the top 10 hit compounds with the highest scores from the MD simulations. 4

5

Figure S5 Interaction diagram of the top 10 compounds with the highest scores from MD simulations at the PAK4 active site. 6

Figure S6 Superimposition diagram of the top 9 hit compounds after R-group screening. 6

Figure S7 Structural comparison of Compd 26 during the MD_3 simulation process. 7

Figure S8 Superimposition diagram of Compd 26 and the reference compound Compd 55 at the PAK4 active site. 7

Figure S9 The docking poses of Compd 26 in PAK1 and PAK4 using the XP protocol of Schrödinger's Glide module. 8

Supplementary Tables

Table S1 Chemical Structures and IC_{50} values of the active compounds. 8

Table S2 Binding free energy contributions calculated using the MM/GBSA method following the process of cross-docking. 10

Table S3 The top 9 hit compounds after R-group screening.

11

Table S4 Analysis of hydrogen bond interactions.

Table S5 Binding free energy contributions for the Compd 26-7CP4 complex. 12

13

Table S6 The docking score results of Compd 26 against PAK4 and PAK1**.**

13

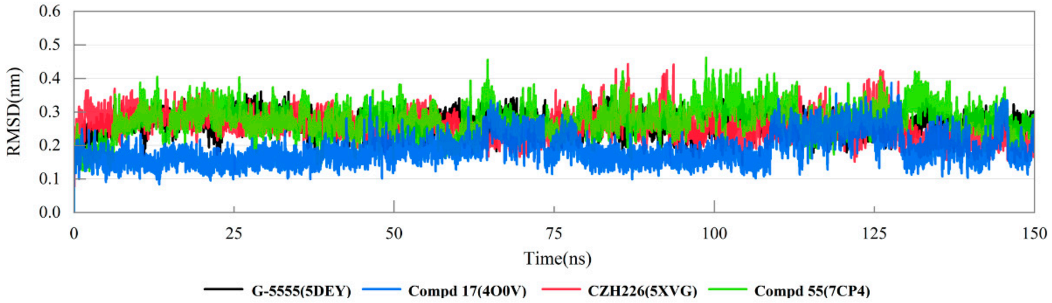

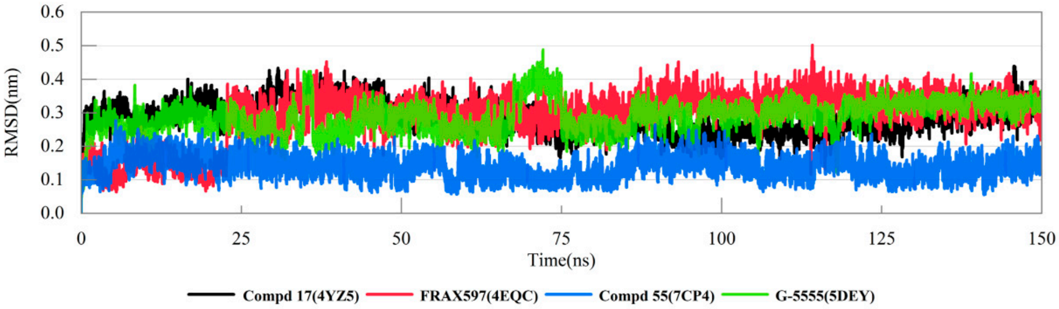

Figure S1. RMSD analysis of co-crystal inhibitors during MD simulations after cross-docking. The top panel shows PAK4 complexes with G-5555 (black), Compd 17 (blue), CZH226 (red), and Compd 55 (green). The bottom panel shows PAK1 complexes with Compd 17 (black), FRAX597 (red), Compd 55 (blue), and G-5555 (green).

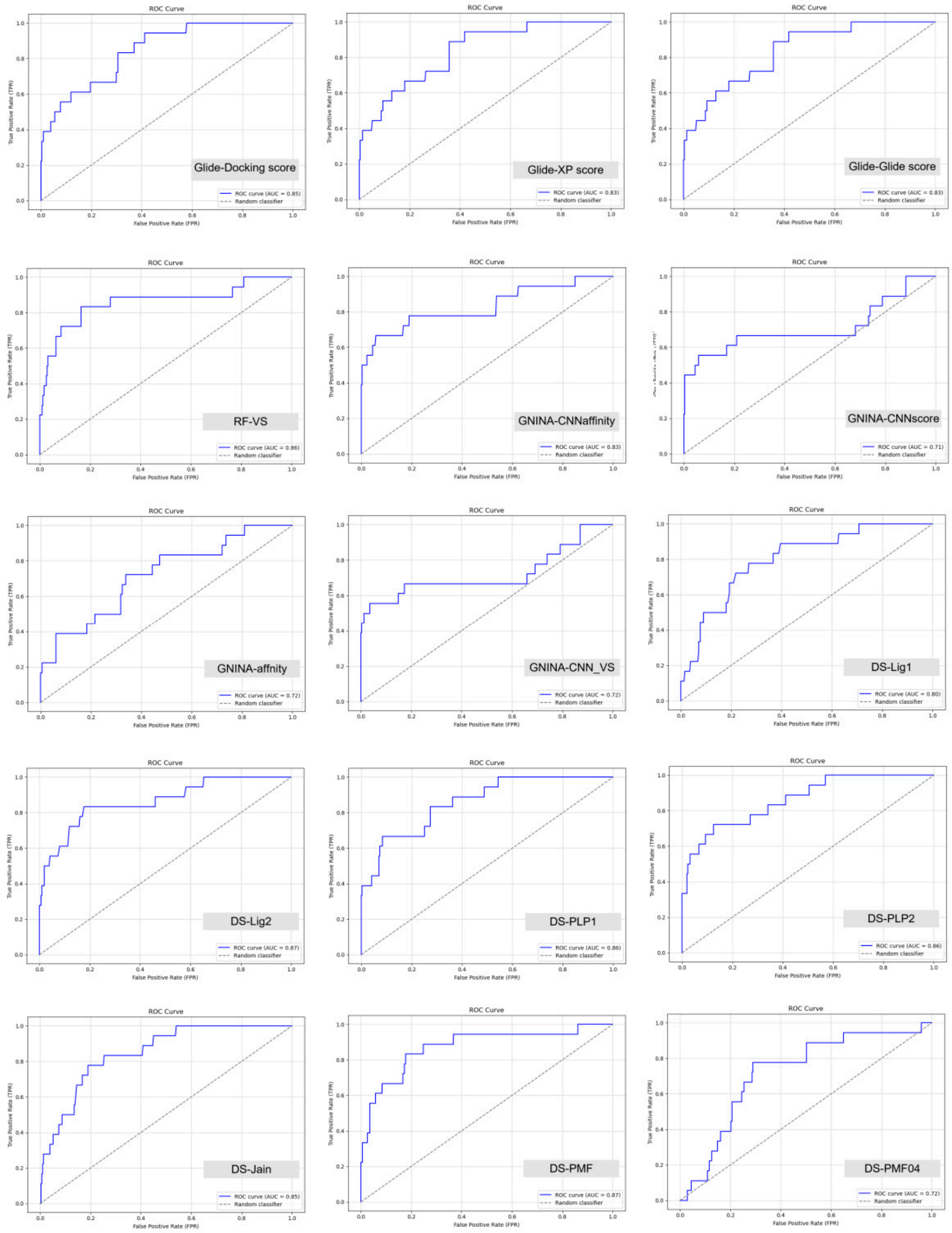

Figure S2. ROC curves for the scoring functions

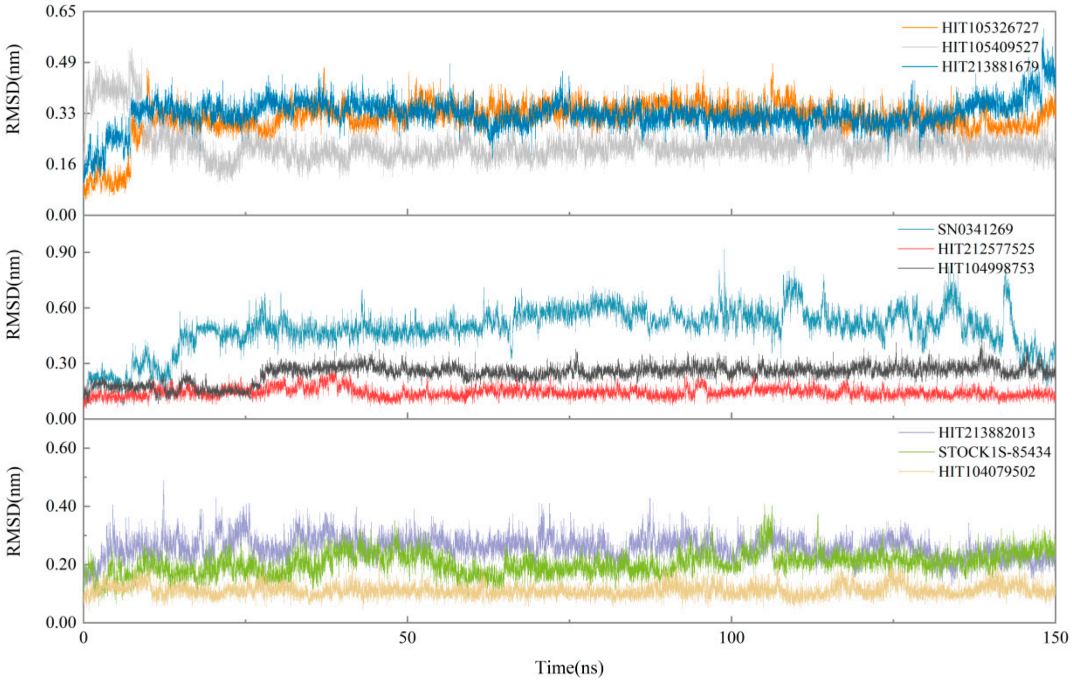

Figure S3. RMSD analysis of the top 10 hit compounds during MD simulations excluding STOCK7S-56165.

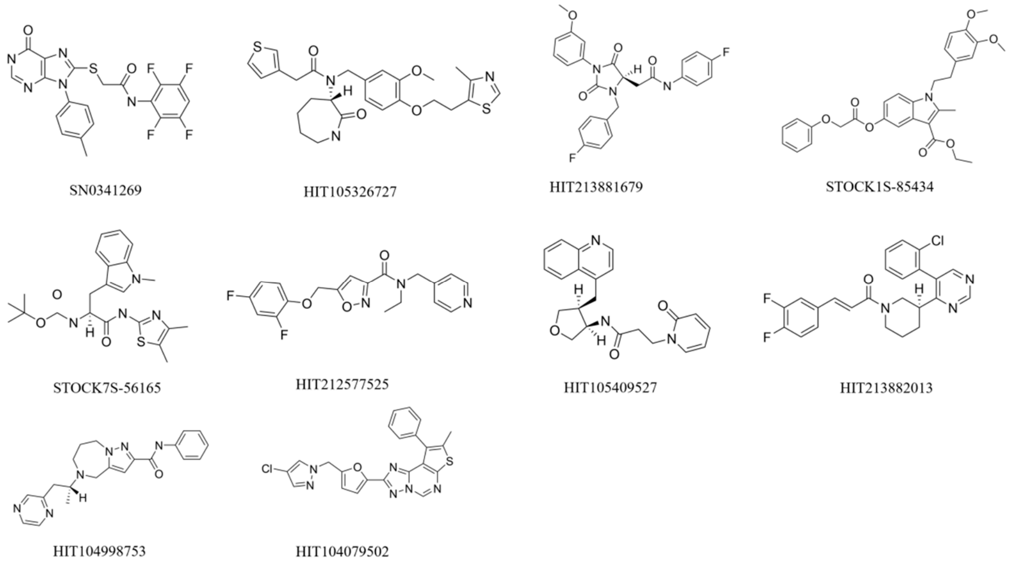

Figure S4. Structures of the top 10 hit compounds with the highest scores from MD simulations.

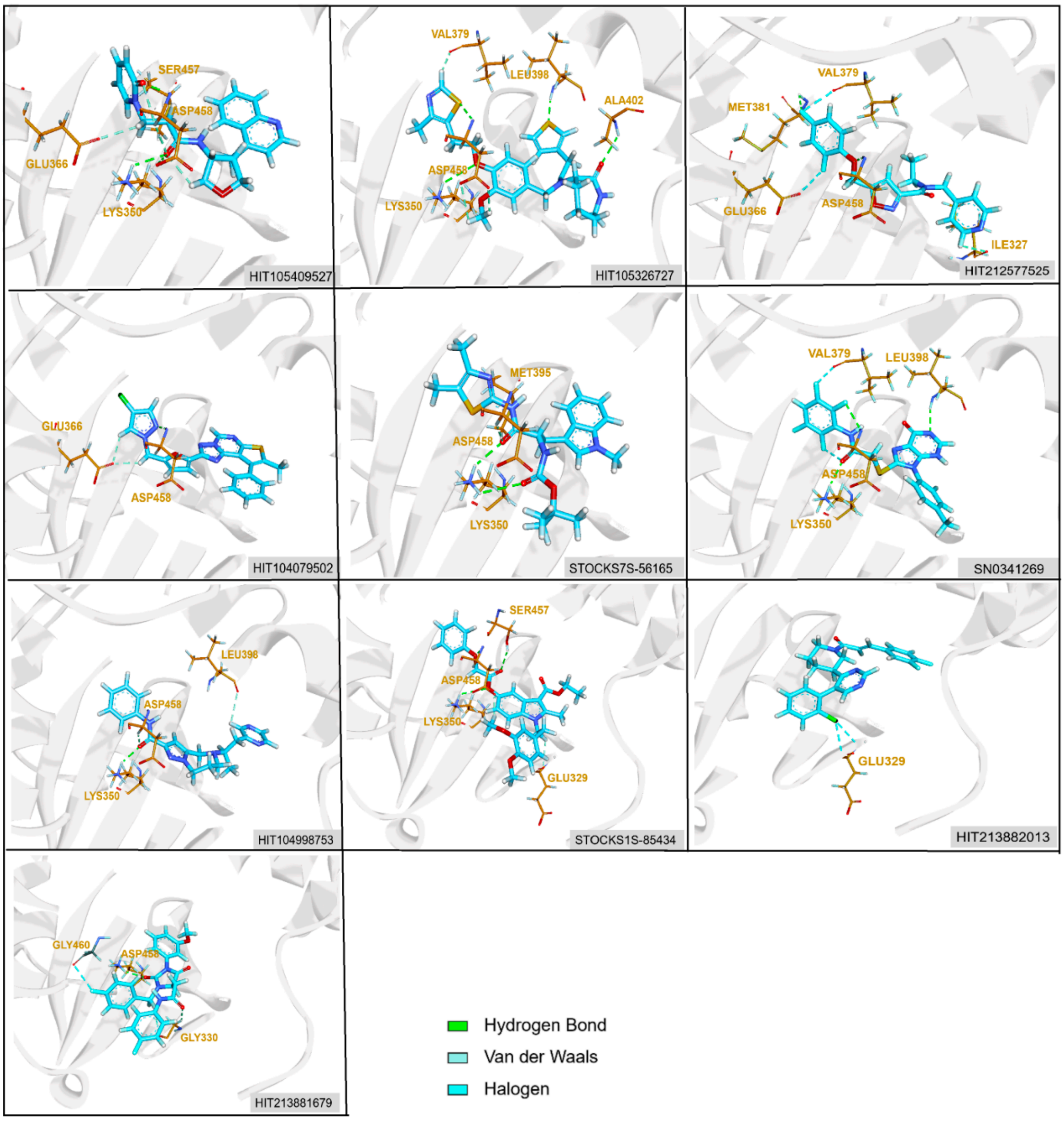

Figure S5. Interaction diagram of the top 10 compounds with the highest scores from MD simulations at the PAK4 active site (PDB ID: 7CP4)

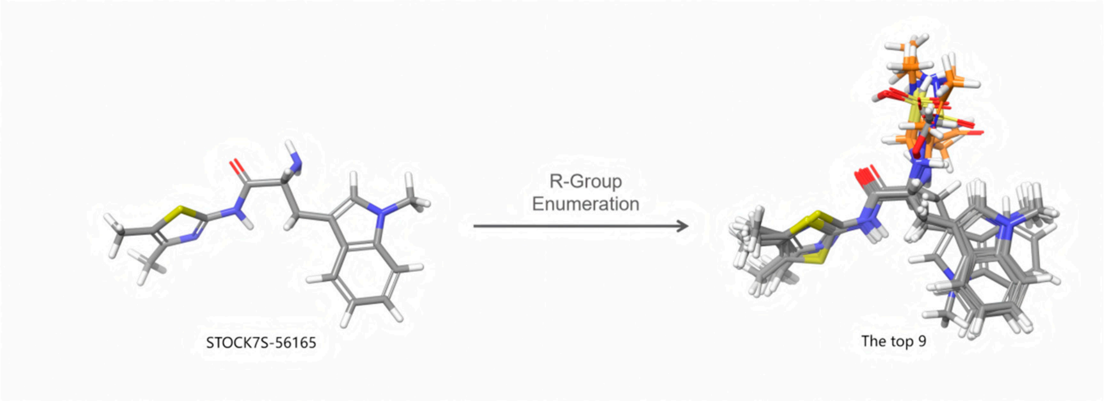

Figure S6. Superimposition diagram of the top 9 hit compounds after R-group screening at the PAK4 active site (PDB ID: 7CP4).

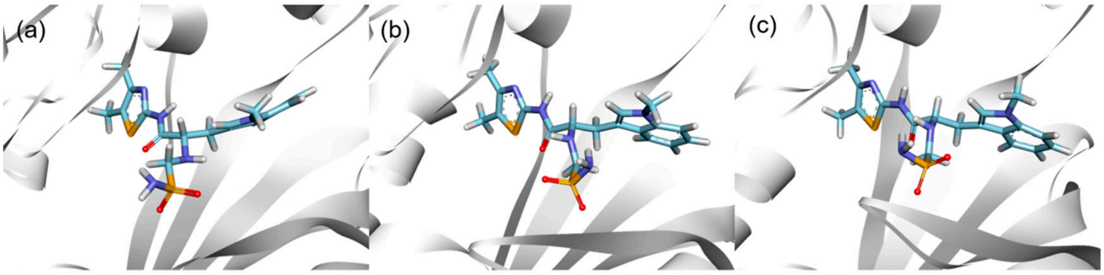

Figure S7. Structural comparison of Compd 26 during the MD_3 simulation process: (a) 1 ns; (b) 100 ns; (c) 250 ns.

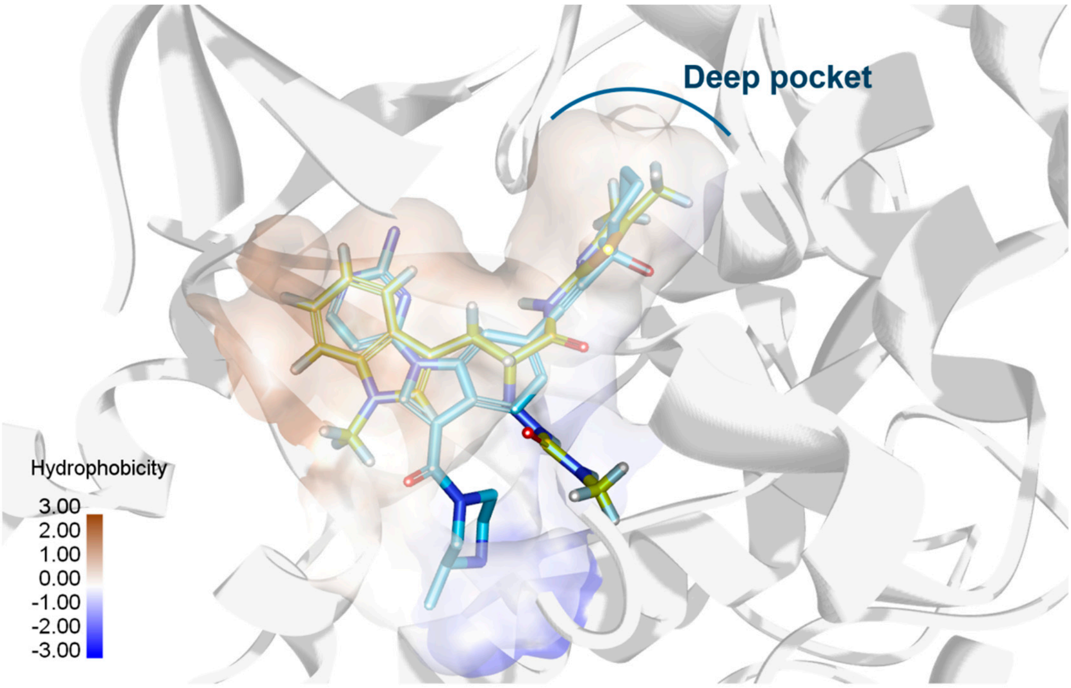

Figure S8. Superimposition diagram of Compd 26 and the reference compound Compd 55 at the PAK4 active site (PDB ID: 7CP4) after docking using the XP protocol of Schrödinger's Glide module , with yellow representing Compd 26 and blue representing Compd 55. The transparent surface represents the hydrophobic or hydrophilic nature of the active pocket.

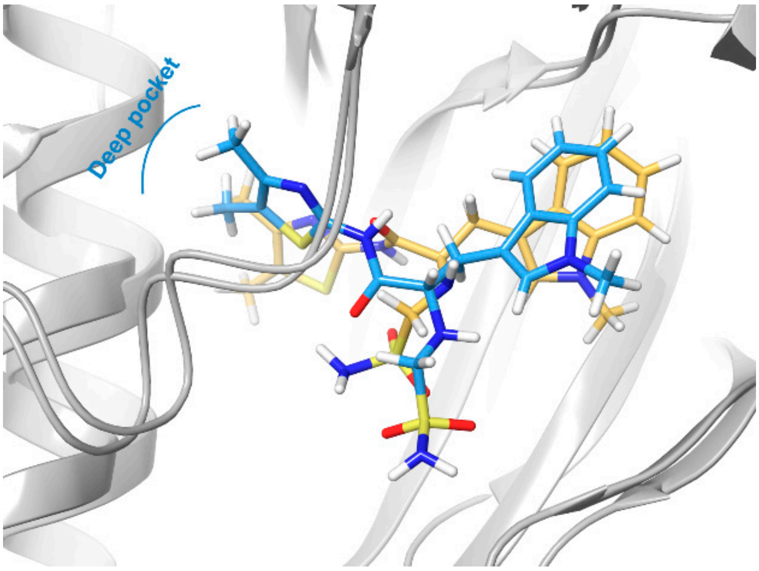

Figure S9. The docking poses of Compd 26 in PAK1 and PAK4 using the XP protocol of Schrödinger's Glide module, with blue representing the pose in PAK4 (PDB ID: 7CP4) and yellow representing the pose in PAK1 (PDB ID: 5DEY).

Table S1. Chemical Structures and IC_{50} values of the active compounds.

Compounds	Chemical Structures	Activities	PDB ID
Compd 55	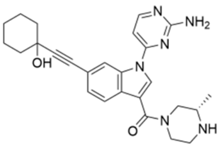	K_i=10.2 nmol/L	7CP4
Compd 47	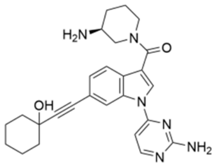	K_i=3.4 nmol/L	7CP3
Compd 41	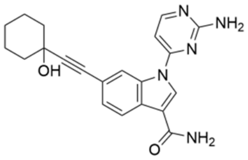	K_i=14 nmol/L	7CMB
CZH226	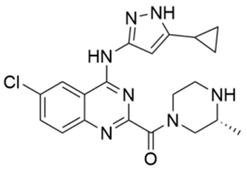	K_i=9 nM	5XVG

CZH216	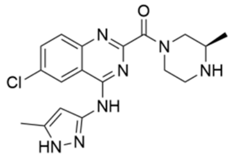	K_i=51 nM	5XVA
Compd 3	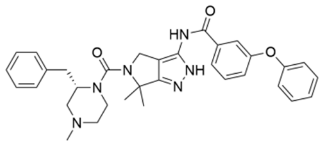	K_i=0.064 uM	4APP
CGP74514A	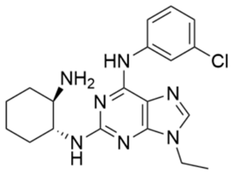	Activity=56% at 10 uM	2CDZ
Compd 13	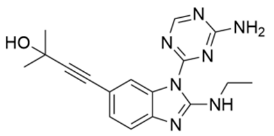	K_i=0.06 uM	4O0Y
Compd 8	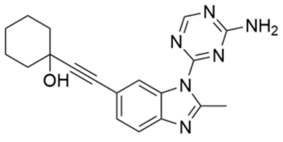	K_i=0.068 uM	4O0X
Compd 17	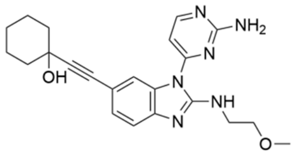	K_i=3.3 nM	4O0V
KY-04031	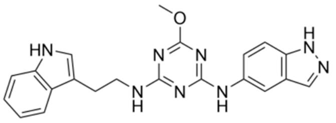	K_i=0.79 ± 0.05 uM	4NJD
PF-3758309	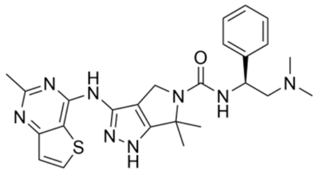	K_i=0.33 ± 0.15 uM	2X4Z
CZg353	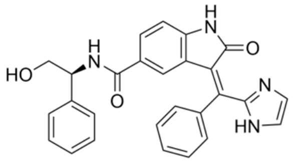	IC_{50}=25 nM	5ZJW

10A	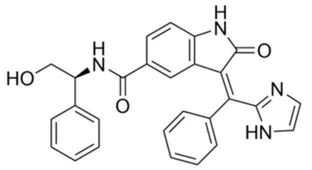	IC_{50}=25nM
LCH-7749944	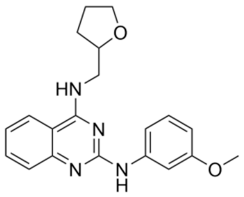	IC_{50}=14.5uM
9D	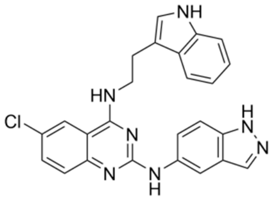	IC_{50}=0.033uM
SPU-106	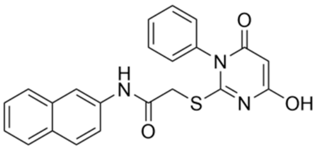	IC_{50}=21.36uM
Compd 12g	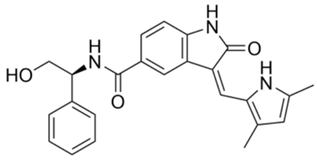	IC_{50}=0.83uM

Table S2. Binding free energy contributions calculated using the MM/GBSA method following the process of cross-docking (energy unit: kcal/mol).

Complexes		ΔG_{vdw}	ΔG_{ele}	ΔG_{solv}	ΔG_{total}
**PAK1**	G-5555 (reference)	-52.28	-39.58	52.33	-57.24
**(**5DEY)	CZH-226	-54.29	-17.42	32.27	-45.31
	Comp 17 (4O0V)	-53.87	-16.75	33.24	-43.32
	Compd 55	-50.21	-25.72	39.18	-42.64
**PAK4**	Compd 55 (reference)	-63.58	-30.26	39.99	-60.81
**(**7CP4)	G-5555	-61.73	-17.07	39.25	-46.6
	FRAX597	-64.75	-13.01	29.68	-55.17
	Compd 17 (4ZY5)	-52.6	-20.21	37.81	-40.3

Table S3. Docking scores and binding free energies of the top 9 hit compounds after R-group screening (energy unit: kcal/mol).

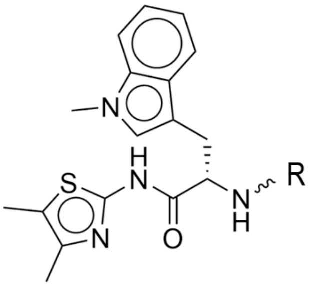

	R	Glide score	ΔG_{total}[1]
Compd 21	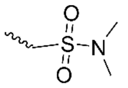	-9.7	-50.93
Compd 23	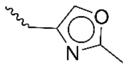	-9.175	-49.97
Compd 24	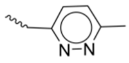	-9.165	-42.35
Compd 25	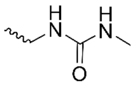	-9.150	-50.95
Compd 26	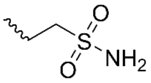	-9.134	-54.06
Compd 22	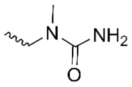	-9.085	-37.94
Compd 28	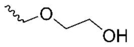	-8.873	-49.96
Compd 29	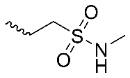	-8.774	-46.80
Compd 27	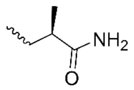	-8.551	-49.85

[1] After 75 ns of molecular dynamics simulations, the binding free energy for 1000 frames was calculated by selecting the stable segments from 50 to 70 ns.

Table S4 Analysis of hydrogen bond interactions.

Complex	Residue	Hydrogen bonds	Occupancy (%)
**Compd 55-7CP4**	459PHE	459F-N-H…compd55-O34	97.593
	398LEU	398L-N-H…compd55-N29	89.961
	398LEU	Compd 55-N30-H…398L-O	82.854
	366GLU	Compd 55-O34-H…366E-O	82.588
	366GLU	Compd 55-O34-H…366E-O	16.866
**STOCK7S-56165-7CP4**	457SER	STOCK-N5-H…457S-O	76.028
	458ASP	458D-N-H…STOCK-N5	56.883
	458ASP	458D-N-H…STOCK-N3	52.497
	350LYS	350K -N-H…STOCK-O19	18.532
	457SER	457S-O-H…STOCK-O18	17.459
**Compd 26-7CP4**	457SER	Compd 26-N5-H…457S-O	94.387
	458ASP	Compd 26-N15-H…458D-O	56.71
	458ASP	Compd 26-N47-H…458D-O	55.33
	332THR	332T-N-H…compd26-O45	51.323
	458ASP	458D-N-H…compd26-N3	47.044
	458ASP	Compd 26-N15-H…458D-O	36.378
	332THR	332T-O-H…compd26-N47	34.104
	350LYS	350K-N-H…compd26-O19	28.318
	458ASP	Compd 26-N15-H…458D-O	26.025
	331SER	331S-O-H…compd26-N47	22.185
	458ASP	458D-N-H…compd26-N5	21.512
	333GLY	338X-N-H…compd26-O45	16.279
	332THR	332T-N-H…compd26-O46	12.886

Table S5. Binding free energy contributions for the Compd 26-7CP4 complex, calculated using the MM/GBSA method from three replicate samples (energy unit: kcal/mol).

	ΔG_{vdw}	ΔG_{ele}	ΔG_{solv}	ΔG_{total}
**MD_1**	-57.45	-64.18	58.63	-63
**MD_2**	-61.85	-40.03	37.61	-64.27
**MD_3**	-57.29	-69.98	61.27	-66
**Compd 55**	-63.58	-30.26	39.99	-60.81

Table S6. The docking score results of Compd 26 against PAK4 and PAK1 using the XP protocol of Schrödinger's Glide module.

**PAK4** (P**DB ID: 7CP4)**		**PAK1 (PDB ID: 5DEY)**	
conformation 1	-9.134	conformation 1	-6.637
conformation 2	-7.989	conformation 2	-6.006
conformation 3	-7.896	conformation 3	-5.986
conformation 4	-7.879	conformation 4	-5.894
conformation 5	-7.858	conformation 5	-5.797
conformation 6	-7.244	conformation 6	-5.795
conformation 7	-7.223	conformation 7	-5.613
conformation 8	-7.2	conformation 8	-5.577
